# A dataset of ground-dwelling nocturnal fauna for object detection and classification

**DOI:** 10.1016/j.dib.2024.110537

**Published:** 2024-05-17

**Authors:** Yassine Sohbi, Jean-Marc Teulé, Alexandre Morisseau, Lola Serrée, Corentin Barbu, Antoine Gardarin

**Affiliations:** a UMR Agronomie, INRAE AgroParisTech, Université-Paris-Saclay, 22 place de l'agronomie, bâtiment F, F-91123 Palaiseau, France; b UMR ECOBIOP 1224 UPPA-INRAE, Aquapole INRAE, 173, RD 918, route de Saint-Jean de Luz, 64319 Saint-Pée sur Nivelle, France; c Chambre d'agriculture Pays de la Loire, 9 rue André-Brouard, CS 70510, 49105 Angers, France

**Keywords:** Multi-class recognition, Ground-dwelling nocturnal fauna, Biodiversity, Deep learning, Mask-RCNN, Computer vision

## Abstract

The exploration of ground-dwelling nocturnal fauna represents a significant challenge due to its broad implications across various sectors, including pesticide management, crop yield forecasting, and plant disease identification. This paper unveils an annotated dataset, BioAuxdataset, aimed at facilitating the recognition of such fauna through field images gathered across multiple years. Culled from a collection exceeding 100,000 raw field images over a span of four years, this meticulously curated dataset features seven prevalent species of nocturnal ground-dwelling fauna: carabid, mouse, opilion, slug, shrew, small-slug, and worm. In instances of underrepresented species within the dataset, we have implemented straightforward yet potent image augmentation techniques to enhance data quality. BioAuxdataset stands as a valuable resource for the detection and identification of these organisms, leveraging the power of deep learning algorithms to unlock new potentials in ecological research and beyond. This dataset not only enriches the academic discourse but also opens up avenues for practical applications in agriculture, environmental science, and biodiversity conservation.

Specifications Table

**Table:** 

Subject	Ecology, Agronomy and Crop Science, Computer Vision, Deep Learning
Specific subject area	The subject area revolves around the application of deep learning techniques for object detection and classification, specifically targeting ground-dwelling nocturnal fauna. This research aims to enhance biodiversity studies by creating and utilizing an annotated dataset, BioAuxdataset, derived from extensive field images. This dataset facilitates the identification of various nocturnal species, leveraging the capabilities of deep learning algorithms to improve understanding and conservation efforts in ecological environments.
Data format	Bioauxdataset is available in the Pascal VOC-XML format
Type of data	JPEG images and XML annotations files
Data collection	BioAuxdataset features 7470 JPEG images and matching XML annotations, covering 8 nocturnal species: carabid, mouse, opilion, slug, shrew, small-carabid, small-slug, worm. Data were captured via custom-built real-time imaging systems using Raspberry Pi nano computers as their core. Each setup includes multiple cameras in varied locations, programmed to snap photos every 15 s, leveraging the Raspberry Pi's ARM processor, 4GB RAM, graphics capabilities, and Wi-Fi connectivity for efficient field operation. Original images, at 3264×2448 resolution, were resized to preserve aspect ratios, resulting in file sizes ranging from 14.7 to 750 KB.
Data source location	Data collection occurred in Grignon, Yvelines, France (15 km west of Versailles; coordinates: 48.849°N, 1.932°E). Storage and processing of the data are handled on a dedicated Linux server at our facility, optimized for image analysis and deep learning simulations.
Data accessibility	Repository name: BioAuxDataset Data identification number: 10.17632/wgsnw4yfgh.1 Direct URL to data: https://data.mendeley.com/wgsnw4yfgh

## 1 Value of the Data

• Researching ground-dwelling nocturnal fauna presents significant challenges due to its wide-ranging implications in domains such as pesticide use management, crop yield prediction, plant disease identification, and biodiversity enhancement through natural predation [[1], [2]].
• The dataset is open access, providing a valuable resource for future researchers and engineers.
• This dataset serves as a foundational tool for training, testing, and validating deep learning algorithms designed to recognize various organisms.

## 2 Data Description

In our research, we concentrated on seven prevalent species of ground-dwelling nocturnal fauna: carabid, mouse, opilion, slug, shrew, small-carabid, small-slug, and worm. The core of our study, the BioAuxDataset, comprises 7470 annotated images, i.e. 7470 JPEG images associated with their corresponding 7470 XML annotation files. A notable distinction exists between our field-acquired images and those typically captured in a laboratory setting. While laboratory images often showcase subjects in full detail, including organ visibility, the fauna in our dataset may be obscured by natural vegetation, situated in dimly lit scenes, or partially outside the frame.

Field images, originally exceeding 3 MB in size, were downsized to facilitate their use without sacrificing quality. The dataset includes both these reduced-size images and augmented versions, varying significantly in size—from the smallest at 14.7 kilobytes to the largest at 2.7 megabytes. This variety ensures that during the training phase, the deep learning model [[3], [4]] encounters a broad spectrum of scenarios, enhancing its performance in subsequent detection tasks.

Furthermore, the dataset's utility for deep learning algorithms is enriched by the number of occurrences of individuals per class and the diversity in the sizes of these individuals, which vary widely among different classes and even within the same class depending on the developmental stage. Such diversity is critical for the model's ability to learn effectively. Table 1 in our study details the occurrences of individuals per class, while Table 2 provides insights into the minimum and maximum sizes of individuals by class, including the ratio of an individual's size relative to the image frame, underscoring the dataset's complexity and richness in teaching the model to recognize and differentiate between various organisms.

**Table 1:** Number of individuals for the 7 classes of BioAuxDataset.

	number of individuals
Carabid	2651
Mouse	683
Opilion	1154
Shrew	763
Slug	3178
small-carabid	1182
Worm	1196

**Table 2:** We can see that the ratio size (here, the image size is the product height*width) of carabid, opilion, small-carabid and small-slug varies from 0.02 % to 0.8 % of the image size.

	minimum size	ratio(%)	maximum size	ratio(%)
carabid	2,760	0.23	28,200	0.56
mouse	18,984	2.53	64,064	4.29
opilion	16,884	0.33	39,360	0.78
shrew	38,150	5.08	850,450	16.87
slug	3,120	0.41	911,790	18.09
small-carabid	1,040	0.02	6,138	0.12
small-slug	248	0.02	19,822	0.39
worm	720	0.09	226,324	30.17

## 3 Experimental Design, Materials and Methods

### 3.1 Experimental devices

**Fig. 1:**
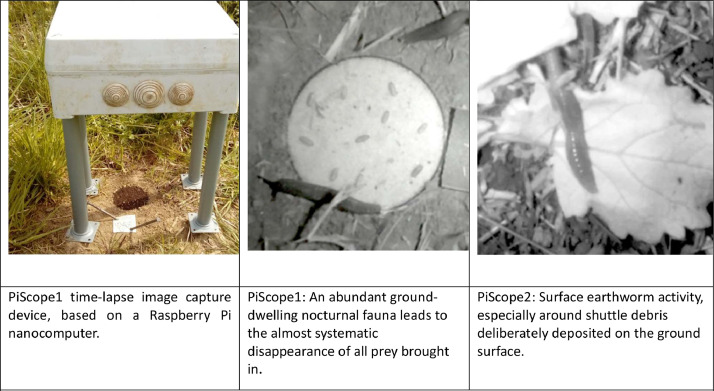
Experimental field devices.

To address the unique requirements of field visualization, we engineered a bespoke real-time image capture system leveraging the compact and versatile Raspberry Pi nanocomputer. Our setup incorporated multiple cameras from Bushnell and Berger Et Shröter, strategically positioned across various plots. Each camera, equipped with an SD memory card, was programmed to automatically capture images at fifteen-second intervals. These devices were managed by the Raspberry Pi, a highly portable nanocomputer boasting an ARM microprocessor, 4GB of RAM, a dedicated video card, and Wi-Fi connectivity, facilitating seamless operation in diverse field conditions. This innovative arrangement ensures continuous, detailed monitoring of our study areas, as depicted in Fig. 1 (image on the left), highlighting the system's field adaptability.

An intuitive web interface simplifies the operation of our system. Daily, the SD card's contents are transferred to one of the hard drives on our image server for storage. Each field-captured JPEG image boasts dimensions of 3264 by 2448 pixels. Our setup included two experimental devices:

• PiScope1 (depicted in Fig. 1, middle image) is a sophisticated image acquisition tool that, from 2016 to 2019, captured over 70,000 raw RGB color images and several videos, providing a rich dataset for analysis.
• PiScope2 (shown in Fig. 1, right image) contributed an additional 33,000+ raw images during the 2017-2018 period. These images, ranging in size from 2 to 3.5 Megabytes, offer detailed insights into the studied phenomena.

To ensure these high-resolution images are ready for analysis, they undergo three stages of processing as shown below:

### 3.2 Image resizing

The initial phase of our image processing involved downsizing the raw images to make them more manageable for computational analysis, without compromising the integrity of the information they contained. This resizing is essential because deep learning algorithms require optimized image sizes for efficient processing in memory or on GPUs. Our approach maintained the original aspect ratio of each image to ensure that the proportions of the subjects within them remained consistent relative to each other. To achieve this, we standardized the width of all reduced images to 1000 pixels. Consequently, the size of these adjusted images now ranges from 14.7 to 750 kilobytes, allowing for a balance between detail preservation and computational efficiency.

### 3.3 Image labeling and augmentation

Following the resizing process, we annotated the images using the LabelImg application, a Python-based tool. In situations where the quantity of certain species was insufficient for effective model training, we generated additional images through image augmentation techniques. These techniques were carefully applied to respect the natural movement patterns of the subjects. For instance, while carabids could rotate in any direction, they always remained grounded. Conversely, shrews, slugs, and mice displayed the ability to climb and potentially adopt vertical positions, reflecting their diverse locomotion capabilities. The inherent elasticity of creatures like slugs and worms necessitated the use of image segmentation for duplication, employing tools such as Gimp for image retouching or the OpenCV library for coding solutions. Additionally, we developed scripts to rotate images, ensuring the original annotations were preserved post-transformation.

This annotation phase was meticulous, governed by strict labeling conventions to facilitate precise model training and, consequently, more accurate organism detection. These conventions included:

• the complete labeling of visible individuals
• the annotation of partially obscured subjects
• the crafting of bounding boxes that snugly encapsulate the subjects.

**Fig. 2:**
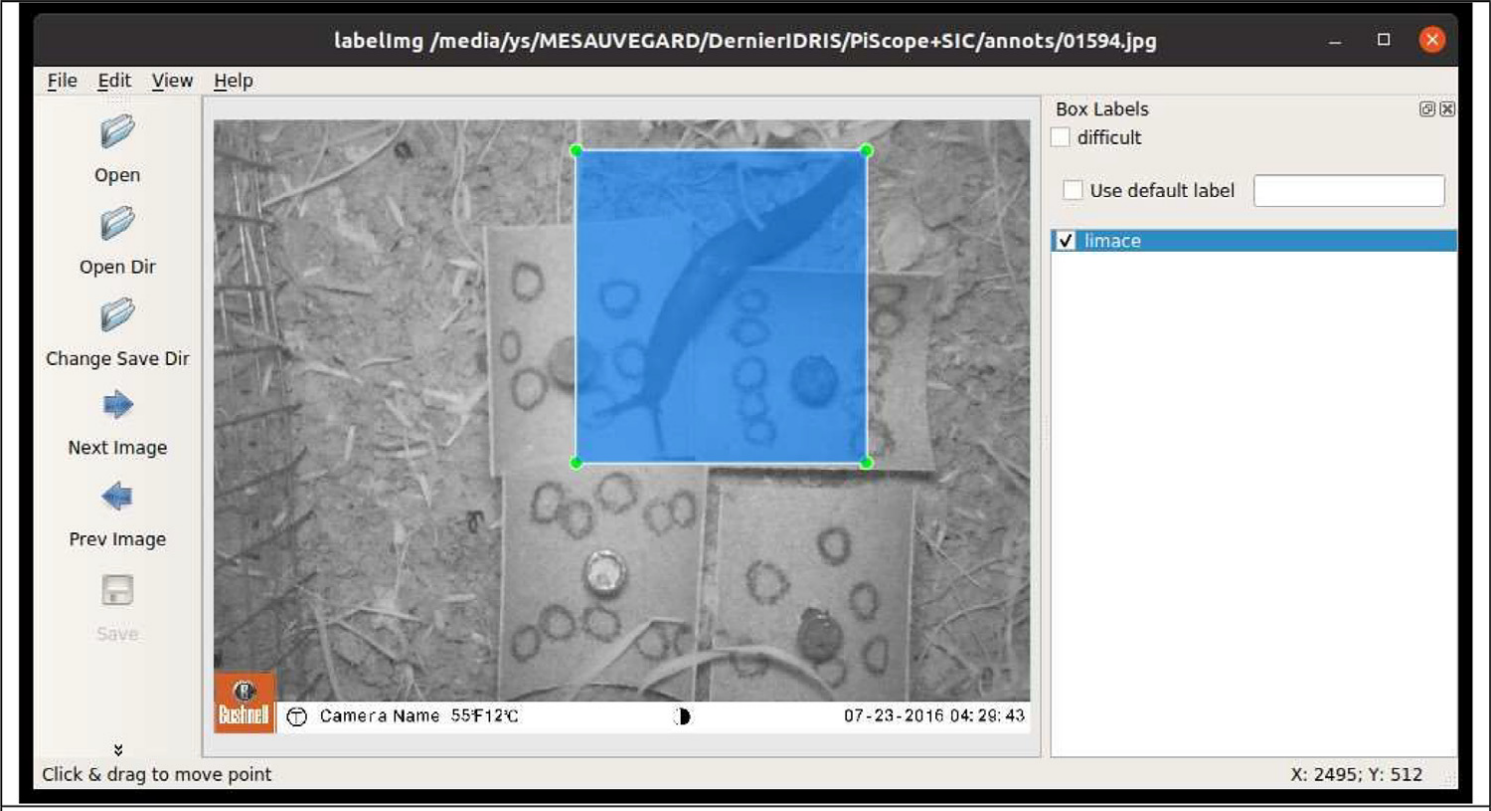
An example of an image annotated with Labelimg, here a slug. The time of acquisition is indicated at the bottom of the image.

Fig. 2. shows an example of an annotated image.

### 3.4 Temporal dynamics of individuals

For the purposes of testing and validating learning models, we have put online 3 new raw image datasets: “2016-6-30-Bushnell-Chrysope”, “2016-7-27-Bushnell-SpidersAnchomenus-Slugs” and “2016-7-13au15-Carnage”. Both datasets contain manual annotation xml files and a sub-directory called "automaticannotation" containing automatic annotation xml files created by a learning model. In addition, in each dataset, a CSV file (metadata.csv) summarizes the results of the automatic annotation. This file contains, among other information, the following columns:

• dataset identifier
• image name
• a column for each class (carabid, ..., worm)
• a presence/absence indicator
• date and time

**Fig. 3:**
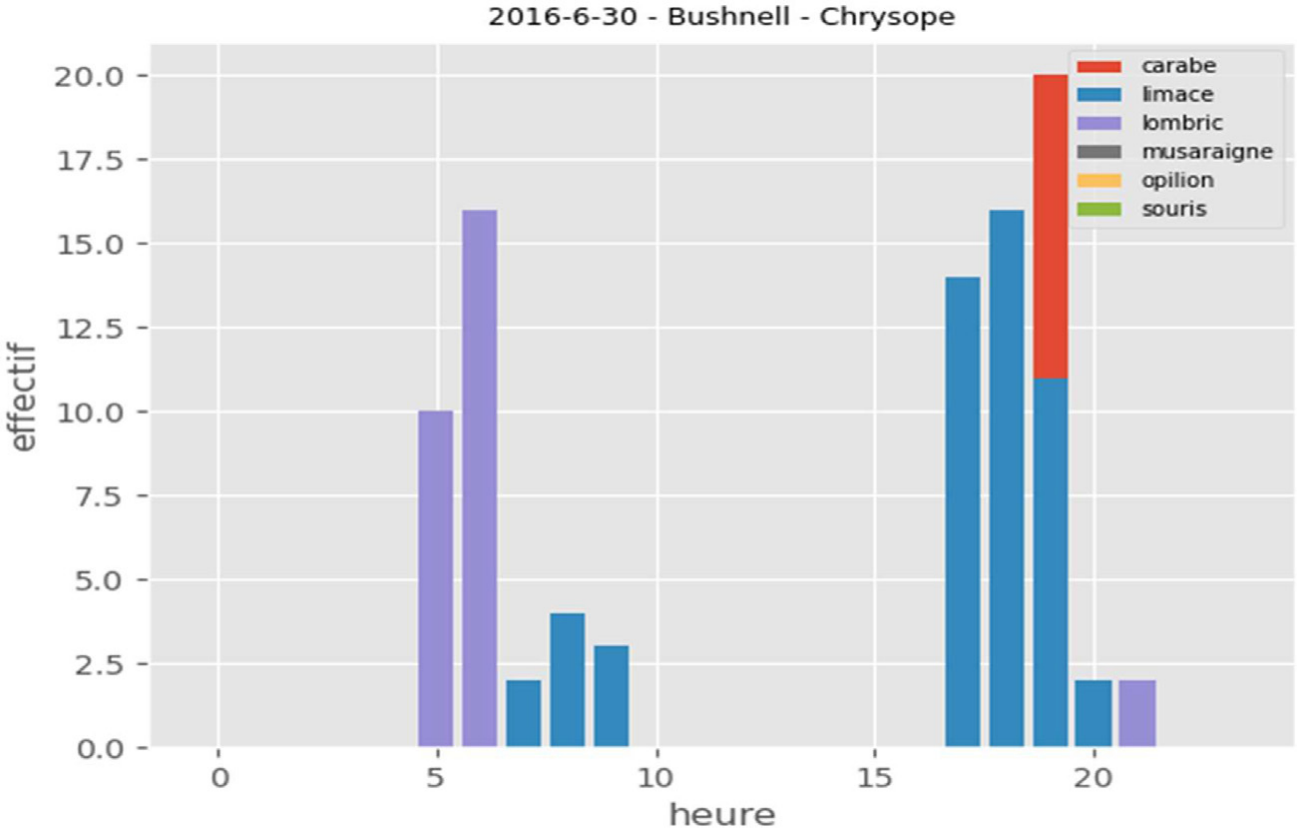
Individuals count of each class over 24 h, dataset: "2016-6-30-Bushnell-Chrysope».

**Fig. 4:**
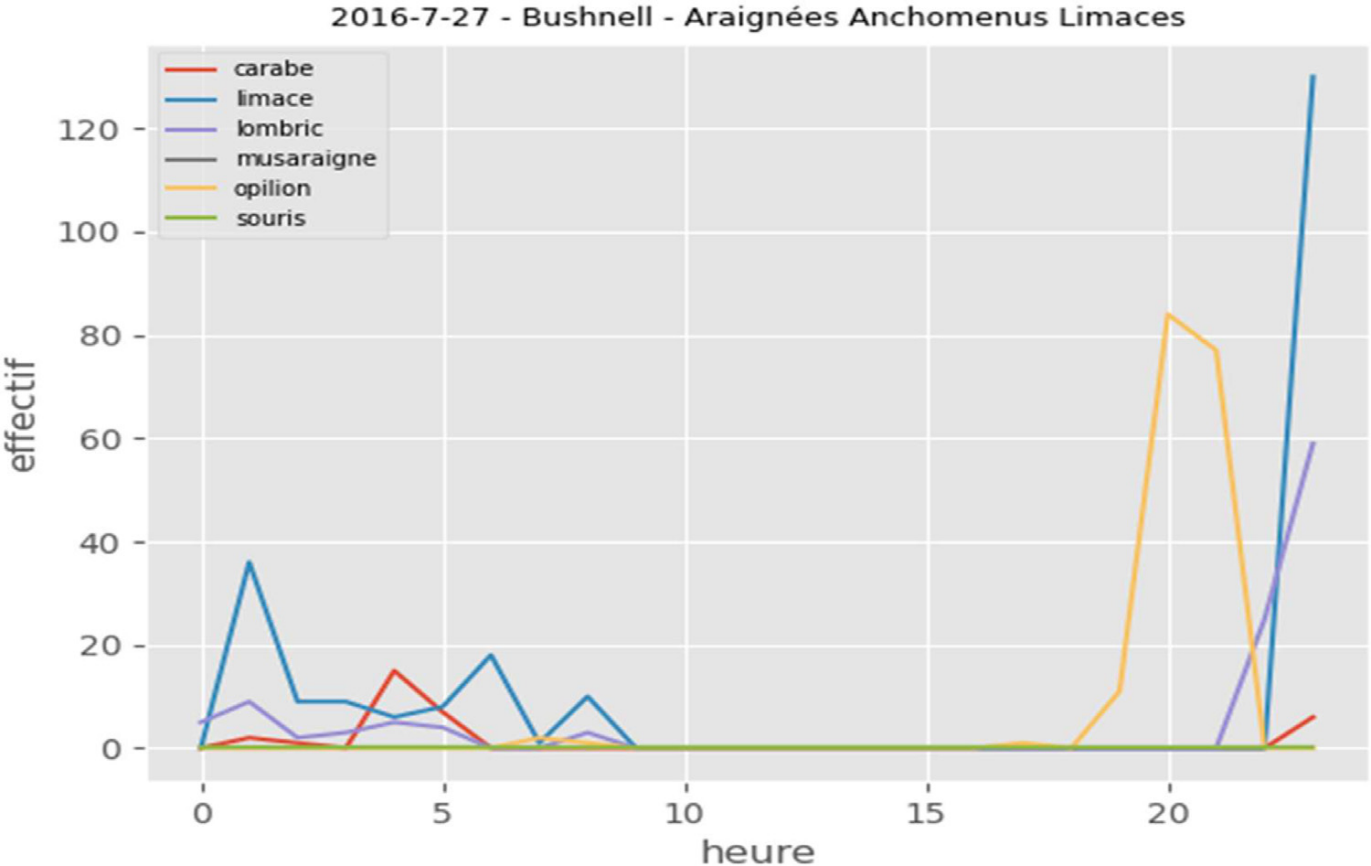
Individual's dynamics over 24 h, dataset "2016-7-27-Bushnell-Anchomenus Spiders Slugs"

This CSV file can be queried for outputs such as the count of individuals over a given period (Fig. 3) or the dynamics (temporality) of individuals (Fig. 4).

## Limitations

None.

## Ethics Statement

The authors have read and follow the ethical requirements for publication in Data in Brief and confirming that the current work does not involve human subjects, animal experiments, or any data collected from social media platforms.

## Credit Author Statement

**Yassine Sohbi:** Annotation, Processing and image augmentation, Methodology, Writing. **Jean-Marc Teulé:** Design and implementation of the experimental device. **Alexandre Morisseau:** Image annotation. **Lola Serrée:** Image annotation. **Corentin Barbu:** Data storage. **Antoine Gardarin:** Funding acquisition, Supervision.

## Data Availability

BioAuxdataset: a dataset of ground-dwelling nocturnal fauna for object detection and classification (Original data) (Mendeley Data)
